# Supplementary material for: Luxury Zinc Supply Prevents the Depression of Grain Nitrogen Concentrations in Rice (Oryza sativa L.) Typically Induced by Elevated CO2
Source: Plants (Basel). 2023 Feb 13;12(4):839. doi: 10.3390/plants12040839 (PMC9963902; doi:10.3390/plants12040839)
Supplement: Supplementary file 1 [file plants-12-00839-s001.zip › plants-2087895-supplementary.pdf]

SUPPLEMENTAL DATA

Table S1. Mean concentrations (n = 3) of a selection of essential elements at panicle emergence in the leaves of IR26 and IR36 grown with marginal and luxury concentrations of Zn at a[CO_2] and e[CO_2].

Treatment	P	K	S	Mg	Ca	Na	Fe	Mn	Cu
	----------------(g kg^{-1})----------------					---------(mg kg^{-1})----------			
IR36 Luxury Zn a[CO_2]	2.51	25.4	3.72	3.56.	7.03	124	130	570	21
IR36 Luxury Zn e[CO_2]	2.60	23.80	3.20	3.80	6.78	179	150	670	19
IR26 Luxury Zn a[CO_2]	2.21	22.4	2.90	2.70	4.63	174	97	240	12
IR26 Luxury Zn e[CO_2]	2.71	24.7	3.06	3.45	4.73	146	120	330	16
IR36 Marginal Zn a[CO_2]	2.42	26.2	3.03	4.00	6.38	163	160	360	24
IR36 Marginal Zn e[CO_2]	2.70	24.1	2.50	4.72	8.07	213	190	709	23
IR26 Marginal Zn a[CO_2]	2.40	25.2	2.53	3.39	4.29	178	110	267	14
IR26 Marginal Zn e[CO_2]	3.00	21.4	2.80	4.62	5.38	118	170	408	15
[1]Minimum sufficient	0.90	10.0	1.80	2.00	1.50	NA	70	150	8

[1]Minimum sufficient concentrations [31,32]. NA = not applicable

Table S2. ANOVA for the treatment effects on grain Zn concentration (see Fig. 5A).

Variable	Sum of squares	Degrees of freedom	MS	F	P
Variety	1281.15	1	1281.15	253.678	0.000000
Zn	1808.74	1	1808.74	358.144	0.000000
CO_2	252.53	1	252.53	50.002	0.000003
Variety*Zn	156.83	1	156.83	31.053	0.000042
Variety*CO_2	18.11	1	18.11	3.587	0.076473
Zn*CO_2	53.55	1	53.55	10.603	0.004955
Variety*Zn*CO_2	44.96	1	44.96	8.903	0.008771
Error	80.81	16	5.05		

Formatte

Table S3. ANOVA for the treatment effects on grain N concentration (see Fig. 5B).

Variable	Sum of squares	Degrees of freedom	MS	F	P
Variety	13.14	1	13.14	16.08	0.001012
Zn	229.67	1	229.67	281.05	0.000000
CO_2	4.57	1	4.57	5.59	0.031044
Variety*Zn	2.59	1	2.59	3.17	0.093934
Variety*CO_2	1.80	1	1.80	2.21	0.156857
Zn*CO_2	0.32	1	0.32	0.39	0.541268
Variety*Zn*CO_2	0.01	1	0.01	0.01	0.929240
Error	13.07	16	0.82		
